# Vascular complications of ProGlide *versus* Prostar in transcatheter aortic valve replacement (TAVR) procedures: meta-analysis

**DOI:** 10.1093/bjsopen/zrad061

**Published:** 2023-07-27

**Authors:** Yuwei Xiang, Chen Chen, Jichun Zhao, Yukui Ma, Bin Huang, Zhoupeng Wu

**Affiliations:** Division of Vascular Surgery, Department of General Surgery, West China Hospital, Sichuan University, Chengdu, China; Division of Cardiology, West China Hospital, Sichuan University, Chengdu, China; Division of Vascular Surgery, Department of General Surgery, West China Hospital, Sichuan University, Chengdu, China; Division of Vascular Surgery, Department of General Surgery, West China Hospital, Sichuan University, Chengdu, China; Division of Vascular Surgery, Department of General Surgery, West China Hospital, Sichuan University, Chengdu, China; Division of Vascular Surgery, Department of General Surgery, West China Hospital, Sichuan University, Chengdu, China

## Abstract

**Background:**

The aim of this study was to compare the vascular complications of ProGlide and Prostar in percutaneous transfemoral transcatheter aortic valve replacement.

**Methods:**

Electronic databases were searched in July 2022 for studies that compared the vascular complications of ProGlide and Prostar for percutaneous closure in transcatheter aortic valve replacement. The primary outcome was major vascular complications and the secondary outcomes were minor vascular complications, types of access-site vascular complications, device failure, and additional intervention. Estimates of relative effects were pooled to generate ORs and their 95 per cent c.i. using a random-effects model. The risk of bias in non-randomized comparative studies was assessed using the Risk Of Bias In Non-randomized Studies - of Interventions (‘ROBINS-I’) tool.

**Results:**

Nine studies were identified and a total of 7529 patients were included. Among them, 4144 patients received ProGlide and 3385 received Prostar. The pooled data showed that the risk of major vascular complications was significantly lower with ProGlide *versus* Prostar (OR 0.50, 95 per cent c.i. 0.32 to 0.78). Regarding the types of vascular complications, vascular trauma was the most common complication and the risk was similar between groups (OR 1.02, 95 per cent c.i. 0.55 to 1.91). ProGlide had a lower risk of bleeding complications (OR 0.46, 95 per cent c.i. 0.22 to 0.94), but a higher risk of ischaemia complications (OR 1.90, 95 per cent c.i. 1.10 to 3.27). The risk of device failure was lower in the ProGlide group (OR 0.45, 95 per cent c.i. 0.21 to 0.95). Both groups had a similar risk of having additional interventions for vascular complications (OR 1.02, 95 per cent c.i. 0.75 to 1.39). The use of ProGlide was associated with a lower risk of additional surgical treatments (OR 0.52, 95 per cent c.i. 0.34 to 0.80), but a higher risk of endovascular treatments (OR 2.69, 95 per cent c.i. 1.29 to 5.63).

**Conclusion:**

In percutaneous transfemoral transcatheter aortic valve replacement procedures, ProGlide has superior safety and efficacy when compared with Prostar; it is associated with fewer major vascular complications and device failures. The vascular complications of ProGlide are more likely to be dealt with using endovascular treatments than surgical treatments.

## Teaser

Although several studies have reported the outcomes of using ProGlide and Prostar during transcatheter aortic valve replacement procedures, the results have been inconsistent. The aim of this study was to integrate the evidence to date and focus on the outcome of vascular complications. The pooled results demonstrate that ProGlide has superior safety and efficacy over Prostar, with decreased risks of major vascular complications, device failures, and additional surgical treatments. ProGlide has a lower risk of bleeding complications, but a higher risk of ischaemia complications. These findings could help clinicians to choose suture-based vascular closure devices, thereby reducing the incidence of vascular complications.

## Introduction

In the past few decades, transcatheter aortic valve replacement (TAVR) with large-bore transfemoral access has been widely performed due to its superior outcomes compared with alternative access^1,2^. Compared with surgical cut down of the femoral artery, percutaneous transfemoral TAVR with vascular closure devices (VCDs) has fewer vascular complications^3^. In TAVR patients, major vascular complications are independently associated with 1-year mortality^4,5^. Therefore, it is important to determine the optimal VCD that results in the fewest vascular complications in TAVR with large-bore transfemoral access.

There are several types of commercially available VCDs for large-bore arteriotomy with considerable rates of success and acceptable rates of vascular complications^6^. Among them, the most commonly used VCDs are two suture-based systems: Perclose ProGlide and Prostar XL (both by Abbott Vascular, Plymouth, MN, USA). Comparisons between ProGlide and Prostar have been reported in several studies; however, the results are inconsistent^7,8^. Currently, there is no clear evidence regarding which suture-based VCD is optimal in terms of vascular complications, and the selection of VCD is mainly based on the preference of the surgeon.

Previously, one systematic review conducted in 2017 compared ProGlide and Prostar for procedures with large-bore transfemoral access, and no significant differences were observed in the rate of vascular complications^9^. In the past 5 years, more research evidence has been published, with the results being inconsistent with the previous review. With more available data, the aim of the current systematic review and meta-analysis was to compare the difference in vascular complications between ProGlide and Prostar in transfemoral TAVR.

## Methods

### Search strategy

This systematic review and meta-analysis was registered in the PROSPERO system (No. CRD42022290571) and conducted in accordance with the PRISMA guidelines^10^. Electronic databases, including Embase, PubMed, and the Cochrane Library, were searched for eligible articles in July 2022. The search strategy used the terms ‘Prostar’, ‘ProGlide’, ‘Perclose’, ‘suture based’, ‘vascular closure’, ‘artery closure’, ‘arteriotomy closure’, ‘aortic valve replacement’, and ‘aortic valve implantation’. Free-text words were used in combination with the Boolean operators ‘AND’ or ‘OR’. The search was limited to papers written in English, and the reference lists of eligible articles were further searched. The detailed search strategy can be found in *Table S1*.

### Study selection and data collection

Articles comparing the procedural vascular complications of two suture-based VCDs in TAVR with large-bore transfemoral access were eligible, including retrospective or prospective observational studies and RCTs. Studies reporting the outcome of a clip VCD, a collagen-based VCD, a gel-based VCD, or only one type of suture-based VCD were excluded. Reviews, letters, editorial materials, studies reporting duplicated populations, and studies without enough data were also excluded.

The titles and abstracts of the articles were independently screened by two authors (Y.X. and C.C.), and the full-length potential eligible articles were reviewed. The data of eligible articles were collected independently by two authors (Y.X. and C.C.), and the matched data in the observational studies were collected if available. Details regarding the study characteristics, patient characteristics, transfemoral sheath size, vascular complications, device failure, and additional intervention were independently collected and recorded in the predetermined data collection form by two authors (Y.X. and C.C.). Disagreements were resolved by consensus after discussion between the two authors or consultation with a third author (B.H.).

The primary outcome was major vascular complications, which were defined according to the Valve Academic Research Consortium (VARC)^11,12^. The secondary outcomes were minor vascular complications, types of access-site vascular complications, device failure, and additional intervention. Minor vascular complications were also defined according to the VARC. The types of access-site vascular complications consisted of vascular trauma (dissection, pseudoaneurysm, fistula, rupture, or perforation), bleeding (access-site bleeding or haematoma), and ischaemia (stenosis, occlusion, or embolism). Device failure was defined as the inability to achieve haemostasis at the arteriotomy site, leading to alternative treatments. Additional interventions were defined as procedures performed for vascular complications when necessary, which consisted of surgical treatments and endovascular treatments. Surgical treatments were open repair for vascular complications, and endovascular treatments mainly included angioplasty and stenting in the targeted vessel.

### Risk-of-bias assessment

The risk of bias in non-randomized comparative studies was assessed using the Risk Of Bias In Non-randomized Studies - of Interventions (ROBINS-I) tool^13^ by two authors (Y.X. and C.C.) independently. Bias due to confounding, selection of participants, classification of interventions, deviations from intended interventions, missing data, measurement of outcomes, and selection of the reported result was assessed.

### Statistical analysis

The data were analysed using R Studio (R version 4.1.1). Meta-analysis was performed using the Mantel–Haenszel method, and data were pooled using the random-effects model owing to the variability in each included study. The *I*^2^ statistic was used to assess the heterogeneity among studies. Outcomes are reported as ORs with their 95 per cent confidence intervals. Publication and reporting biases were evaluated using Egger’s test. Sensitivity analyses were further performed to confirm the stability of pooled results by excluding retrospective or small-sample studies. Studies with fewer than 500 patients were defined as small-sample studies in the current meta-analysis.

## Results

### Search results

**Fig. 1:**
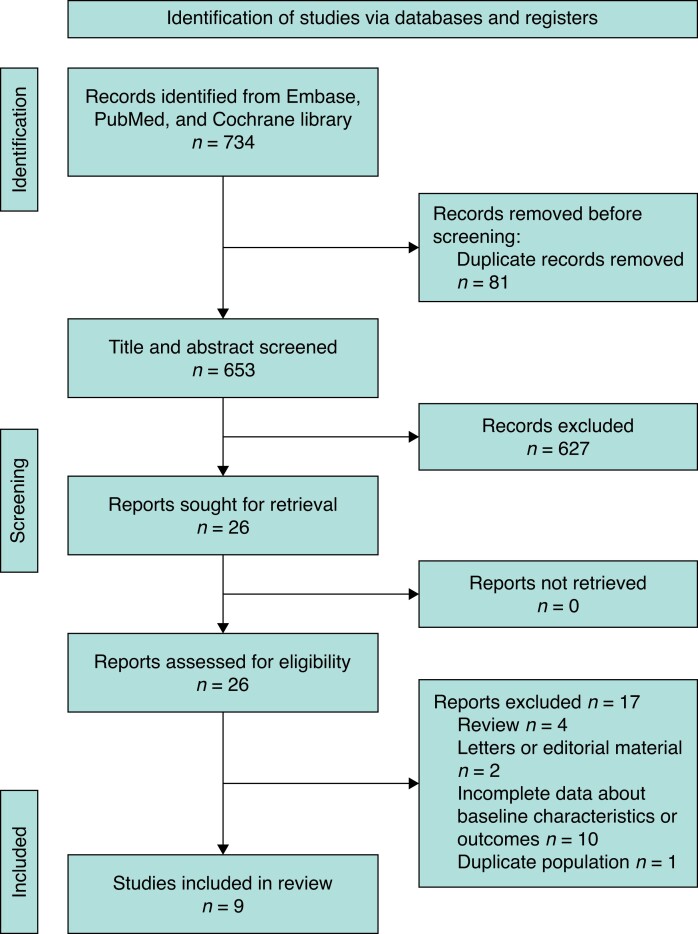
PRISMA flow diagram for the literature search to identify studies of ProGlide *versus* Prostar in percutaneous transfemoral transcatheter aortic valve replacement procedures

A total of 734 potential studies were identified from the database search, 81 of which were excluded as being duplicate records. After title and abstract screening, 26 records were selected for further review. During the full-text review, 17 studies were excluded: six were review articles, letters, or editorial materials^9,14–18^; one reported a duplicated population^19^; and ten provided incomplete data about baseline characteristics or procedure outcomes^20–29^. Finally, nine studies^7,8,30–36^ were included for analysis. The PRISMA flow diagram is shown in *Fig. 1*.

### Study and population characteristics

The nine studies included three retrospective studies, five prospective studies, and one randomized trial. These studies were reported between 2015 and 2022, with sample sizes ranging from 191 to 2583. A total of 7529 patients were included for analysis, of which 4144 received ProGlide and 3385 received Prostar in transfemoral TAVR. The general characteristics of the included studies are shown in *Table 1*.

**Table 1:** Characteristics of included studies comparing the vascular complications of ProGlide and Prostar

First author	Year	Region	Patient source	Study design	ProGlide, *n*	Prostar, *n*
Barbanti	2015	Italy	Ferrarotto Hospital	Single-centre retrospective	125	153
Barbash	2015	Europe, North America, and the Middle East	CONTROL	Multicentre prospective	472	472
Mehilli	2016	Germany	Munich University Clinic and Herzzentrum Bad Segeberg	Multicentre prospective	506	516
Seeger	2016	Germany	University of Ulm	Single-centre prospective	348	237
Dimitriadis	2017	Germany	Herz- und Diabeteszentrum Nordrhein-Westfalen	Single-centre prospective	183	215
Power	2019	Europe and Canada	BRAVO-3	Randomized trial	394	352
Berti	2020	Italy	RISPEVA	Multicentre prospective	1361	1222
Heitzinger	2022	Austria	Medical University of Vienna	Single-centre retrospective	670	112
Marcusohn	2022	Israel	Rambam Health Care Campus	Single-centre retrospective	85	106

The mean age of the included patients was over 80 years and 54.8 per cent were female. Hypertension (81.5 per cent) was the most common combined disease in the pooled cohort, followed by diabetes mellitus (28.7 per cent); peripheral vascular disease (12.9 per cent) was less common. The baseline characteristics and cardiac operative risk stratification were comparable between the ProGlide group and the Prostar group (*Table S2*).

### Quality of the included studies

The risk of bias in the included studies was assessed using the ROBINS-I tool. In most of the included studies, the sheath sizes were similar between groups, but four studies^19,32,33,35^ did not provide detailed information regarding sheath sizes, and were consequently considered to have a moderate risk of bias due to confounding (*Table 2*). The bias due to selection of participants, classification of interventions, deviations from intended interventions, missing data, measurement of outcomes, and selection of the reported result was deemed low in all included studies (*Fig. S1*).

**Table 2:** Sheaths used in the transfemoral procedures of included studies comparing the vascular complications of ProGlide and Prostar

First author	Year	Sheath size (Fr)	
		ProGlide	Prostar
Barbanti	2015	17.8(1.0)	17.7(1.0)
Barbash	2015	18.2(1.7)	18.3(1.7)
Mehilli	2016	18.1(1.0)	17.8(0.8)
Seeger	2016	23.7(1.5)	23.1(4.5)
Dimitriadis	2017	18: 94 (51.3); 19–24: 90 (49.2)	18: 183 (84.7); 19–24: 33 (15.3)
Power	2019	<18: 152 (38.6); 18: 188 (47.4); >18: 52 (13.2)	<18: 95 (27.0); 18: 206 (58.5); >18: 46 (13.1)
Berti	2020	NA	NA
Heitzinger	2022	14: 353 (52.7); 16: 153 (22.8); >16: 154 (23.0)	14: 3 (2.7); 16: 82 (73.2); >16: 26 (23.2)
Marcusohn	2022	15.3(1.0)	14.3(0.8)

Values are mean(s.d) or *n* (%). NA, not available.

### Vascular complications

**Fig. 2:**
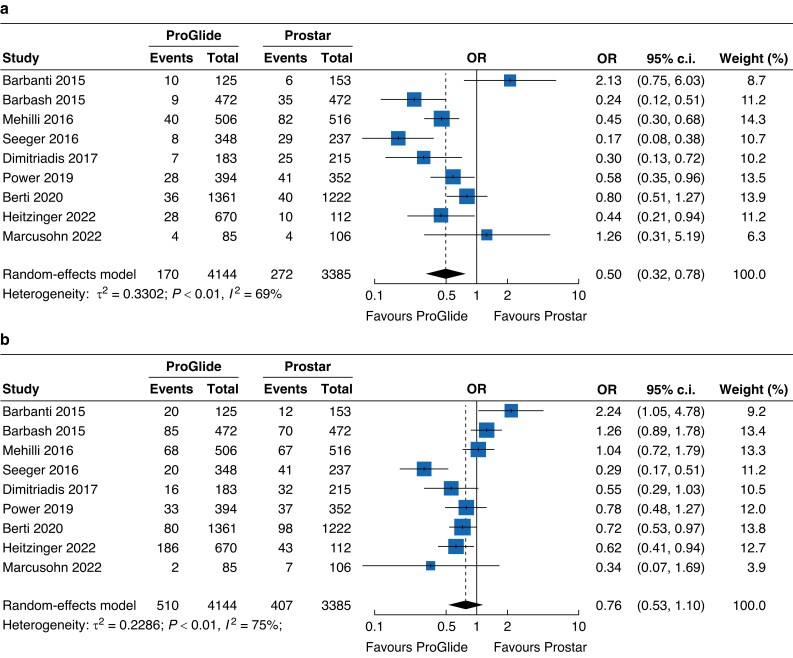
Forest plots of major and minor vascular complications **a** Forest plot of nine studies reporting the risk of major vascular complications. **b** Forest plot of nine studies reporting the risk of minor vascular complications.

All included studies reported data regarding the outcomes of major and minor vascular complications in each group. The pooled data showed a significantly decreased risk of major vascular complications in the ProGlide group compared with the Prostar group, with an OR of 0.50 (95 per cent c.i. 0.32 to 0.78) (*Fig. 2a*). For minor vascular complications, no statistical significance was found between the ProGlide and Prostar groups, with an OR of 0.76 (95 per cent c.i. 0.53 to 1.10) (*Fig. 2b*).

**Fig. 3:**
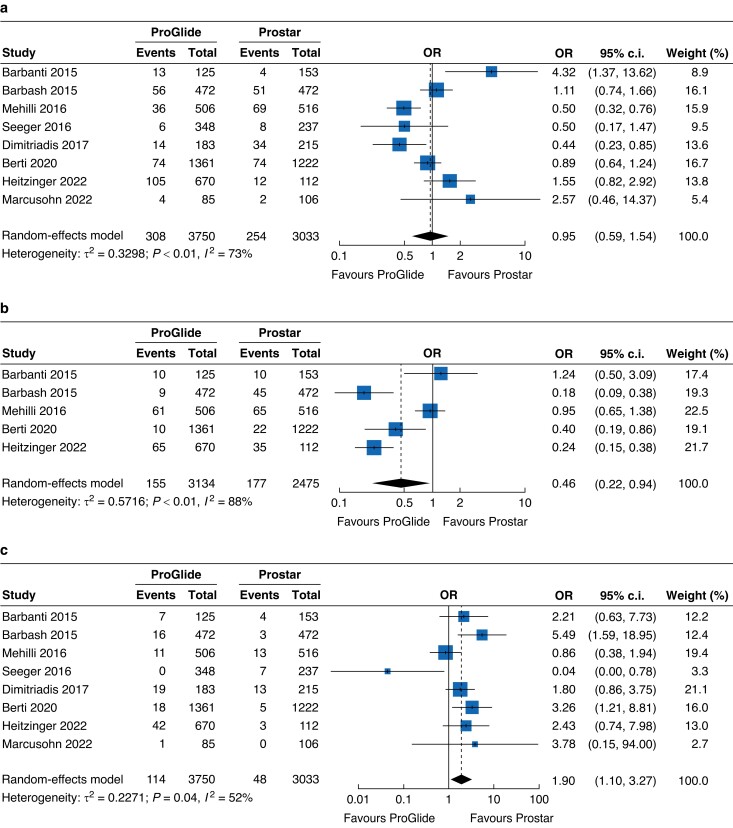
Forest plots of injury, bleeding, and ischaemia complications **a** Forest plot of eight studies reporting the risk of injury complications. **b** Forest plot of five studies reporting the risk of bleeding complications. **c** Forest plot of eight studies reporting the risk of ischaemia complications.

The most common vascular complication was vascular trauma, occurring in 8.3 per cent of patients, with a similar risk in both groups (OR 0.95, 95 per cent c.i. 0.59 to 1.54) (*Fig. 3a*). Among all vascular trauma, no significant difference was found regarding dissection (OR 1.18, 95 per cent c.i. 0.77 to 1.83) or pseudoaneurysm (OR 0.54, 95 per cent c.i. 0.23 to 1.27) between groups (*Fig. S2*). Bleeding complications occurred in 5.92 per cent of all included patients, and the ProGlide group had a significantly lower risk of bleeding complications when compared with the Prostar group (OR 0.46, 95 per cent c.i. 0.22 to 0.94) (*Fig. 3b*). Ischaemia complications also occurred in the whole cohort, affecting 2.4 per cent of patients, and the risk was higher in the ProGlide group (OR 1.90, 95 per cent c.i. 1.10 to 3.27) (*Fig. 3c*). Additionally, in the subgroup analysis, the risk of stenosis was higher in the ProGlide group than in the Prostar group (OR 2.53, 95 per cent c.i. 1.38 to 4.65), whereas no difference was observed regarding occlusion (OR 0.94, 95 per cent c.i. 0.21 to 4.22) (*Fig. S3*).

### Closure device failure and additional intervention

**Fig. 4:**
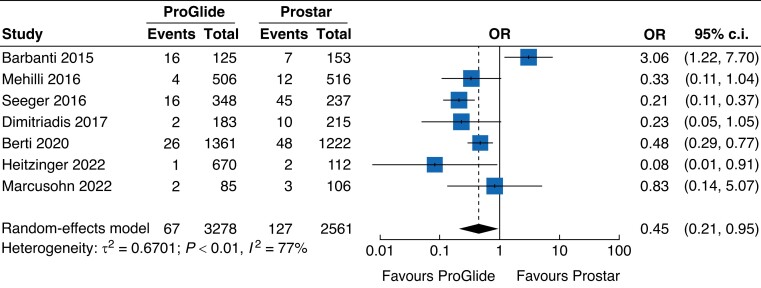
Forest plot of seven studies reporting the risk of device failures

**Fig. 5:**
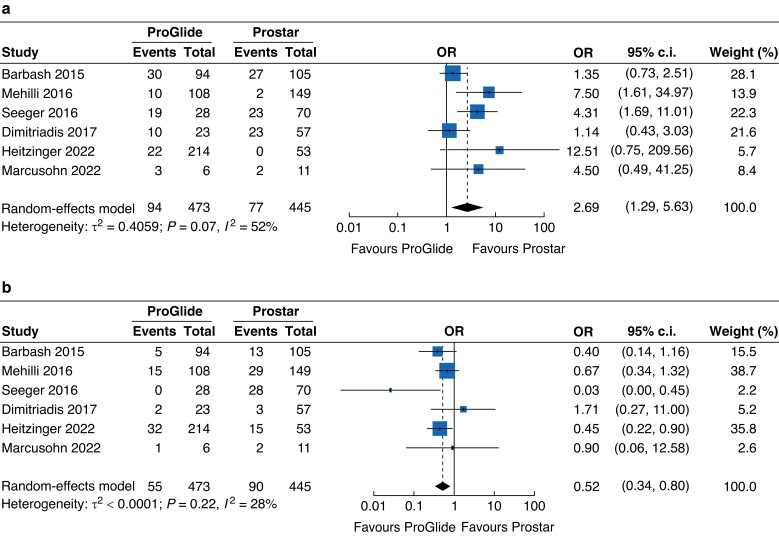
Forest plots of additional endovascular and surgical treatments **a** Forest plot of six studies reporting the additional endovascular treatments for vascular complications. **b** Forest plot of six studies reporting the additional surgical treatments for vascular complications.

According to seven included studies^8,30–32,34–36^, closure device failure occurred on rare occasions in the cohort, with an incidence of 3.3 per cent. When compared with the Prostar group, the ProGlide group had a lower risk of closure device failure, with an OR of 0.45 (95 per cent c.i. 0.21 to 0.95) (*Fig. 4*). According to six studies^7,30–32,35,36^, a total of 316 additional interventions were performed for 918 vascular complications, revealing a similar risk between the ProGlide group and the Prostar group (OR 1.02, 95 per cent c.i. 0.75 to 1.39). In subgroup analysis, the risk of endovascular treatments was higher in the ProGlide group (OR 2.69, 95 per cent c.i. 1.29 to 5.63), whereas this group was associated with a lower risk of surgical treatments (OR 0.52, 95 per cent c.i. 0.34 to 0.80) (*Fig. 5*).

### Publication bias and sensitivity analyses

No obvious publication or reporting bias was found, with *P* values of Egger’s test >0.050. Sensitivity analyses were performed with the exclusion of retrospective or small-sample studies. The primary outcome remained stable after excluding retrospective studies, with an OR of 0.40 (95 per cent c.i. 0.25 to 0.63). This procedure was repeated with the exclusion of small-sample studies, and obtained a very similar result (OR 0.42, 95 per cent c.i. 0.28 to 0.65). Most of the other outcomes were consistent with previous results (*Fig. S4*). The outcome for bleeding complications was consistent after excluding small-sample studies, but did not retain significant difference after the exclusion of retrospective studies. In addition, there was no significant difference in the risk of ischaemia complications between the two groups after the exclusion of retrospective or small-sample studies.

## Discussion

The aim of the present study was to compare the vascular complications in large-bore arteriotomy closure with two suture-based VCDs: ProGlide and Prostar. To the best of the authors’ knowledge, it is the first time the risks of different types of vascular complications have been pooled in a meta-analysis. The included TAVR studies adopted uniform evaluation systems, VARC-2 or VARC-3, to report vascular complications, so that it was possible to derive reliable results from the meta-analysis. The main findings are that, compared with Prostar, ProGlide is a safer device with a decreased risk of major vascular complications; ProGlide has better procedural efficacy with lower risks of device failures and additional surgical treatments; and ProGlide has a lower risk of bleeding complications, but a higher risk of ischaemia complications.

For the outcome of major vascular complications, the majority of the included studies favoured ProGlide^7,30–33,35^. The multicentre prospective study conducted by Barbash *et al*.^7^ identified 472 patient pairs by propensity matching, making this study the only included study at low risk of bias. It provided reliable results, with a higher rate of major vascular complications in the Prostar group (7.4 *versus* 1.9 per cent (*P* < 0.001)). In addition, the recent study with the largest cohort also tended to favour ProGlide, with a propensity-adjusted OR of 0.85 (95 per cent c.i. 0.65 to 1.11)^34^. The studies by Barbanti *et al*.^8^ and Marcusohn *et al*.^36^ demonstrated higher incidences of major vascular complications in the ProGlide group (8.0 *versus* 3.9 per cent (*P* = 0.146) and 4.7 *versus* 3.8 per cent (*P* = 1.000) respectively). However, these results were non-significant, and both studies were single-centre retrospective studies with the smallest and second smallest sample size, which made the results relatively less reliable.

One potential reason for the lower incidence of major vascular complications in the ProGlide group was the lower device failure rate, which was also supported by the pooled evidence in the present study. It is assumed that the different failure rate was due to the different designs of these two devices. The Prostar device delivers four needles simultaneously through the arterial lumen, with any needle malposition leading to device failure. On the other hand, the ProGlide device delivers only two needles that are easier to manipulate, and two ProGlide devices are deployed independently, usually at the 10 and 2 o’clock positions. As a result, the risk of simultaneous failure of both ProGlide devices is low^9^. Insights from the US Food and Drug Administration (FDA) Manufacturer and User Facility Device Experience reported 827 and 175 device failures of ProGlide and Prostar respectively. Only 12.7 per cent of the ProGlide failures were due to failed deployment, compared with 25.1 per cent for Prostar, indicating that it was easier for ProGlide to deploy successfully^20^.

In the current study, most of the included studies presented similar sheath sizes between groups, but, due to inadequate data, a subgroup analysis of sheath size could not be implemented. Based on recent studies, it is still uncertain whether the sheath size is associated with vascular complications. Some studies have reported that the sheath size is an independent predictor of major vascular complications^37,38^, whereas other studies have demonstrated that the difference in sheath diameter has no significant effect on the incidence of vascular complications^39,40^. Thus, RCTs are needed to provide further evidence.

The risk of different types of vascular complications of these two VCDs was first reported in a meta-analysis. The pooled results demonstrated that ProGlide was associated with fewer bleeding complications, but more ischaemia complications. Although the results were unstable in the sensitivity analyses, the potential trends were still clear. The reasons for these phenomena have rarely been discussed in previous studies and need further investigation. The fewer bleeding complications in the ProGlide group were potentially related to the lower device failure rate. For ischaemia complications, although the incidence was relatively low, there were more stenoses in the ProGlide group. Previous studies indicated that the interference of both sutures of independently deployed ProGlide may contribute to stenosis^36,41^. The manufacturer’s recommended ProGlide deployment is to use two sutures at the 2 and 10 o’clock positions, creating an ‘X’-shaped closure, which might cause suture entanglement and subsequent artery stenosis.

The present study found no significant difference in the risk of overall additional interventions, but the types of interventions varied between groups. Most vascular complications in the ProGlide group were treated using endovascular treatments, whereas surgical treatments were performed to deal with vascular complications in the Prostar group. This finding was similar to the report of post-marketing surveillance data from the FDA Manufacturer and User Facility Device Experience database^20^. The data showed that, although surgical interventions were commonly performed in both groups, 82.1 per cent of the adverse events in the Prostar group needed surgical repair, which was much higher than the 54.1 per cent of the ProGlide group. Additionally, 39.3 per cent of the adverse events in the ProGlide group could be treated with manual compression, but, in the Prostar group, it was only 5.3 per cent. One hypothesis was that when one of the ProGlide devices fails, the other one might still work because the two devices are deployed independently, which could limit the severity of events and reduce the need for surgical treatments.

There was one previous systematic review that compared ProGlide and Prostar in percutaneous transfemoral procedures, and no significant differences were observed in the rate of overall vascular complications^9^. This review was conducted in 2017, and only four TAVR studies were included for meta-analysis^7,8,31,42^. In comparison, the present meta-analysis incorporated five more studies published after 2017, including the study with the largest cohort^34^ and the updated data from the population reported by Jochheim *et al*^42^. Thus, the present study demonstrated different results. In addition, the previous systematic review included a study on percutaneous endovascular aortic aneurysm repair (p-EVAR), but the p-EVAR study used an inconsistent definition of vascular complications with TAVR that increased the heterogeneity^27^. The results of the current study might be applicable to percutaneous thoracic endovascular aortic repair (TEVAR) and p-EVAR, which have a similar femoral artery procedure to TAVR.

This study also has some limitations. First, although most of the included studies are prospective studies, randomized trials are still rare, which limits the level of evidence. Second, due to inadequate data regarding sheath sizes, sheath to iliofemoral artery ratio, and arterial calcification in the included studies, the pooled results could not be adjusted according to these factors.

In percutaneous transfemoral TAVR procedures, ProGlide has superior safety and efficacy over Prostar, with lower risks of major vascular complications, device failures, and additional surgical treatments. In addition, ProGlide is related to fewer bleeding complications, but more ischaemia complications.

## Supplementary Material

zrad061_Supplementary_Data

## Data Availability

The data that support the findings of this study are available from the authors upon reasonable request.
