# Supplementary material for: Vascular complications of ProGlide versus Prostar in transcatheter aortic valve replacement (TAVR) procedures: meta-analysis
Source: BJS Open. 2023 Jul 27;7(4):zrad061. doi: 10.1093/bjsopen/zrad061 (PMC10373905; doi:10.1093/bjsopen/zrad061)
Supplement: zrad061_Supplementary_Data [file zrad061_supplementary_data.zip › Supplementary_Material.docx]

**TItle**

**Vascular complications of ProGlide versus Prostar in TAVR procedures: meta-analysis**

Authors

Yuwei Xiang^1^#, MD; Chen Chen^2^#, MD; Jichun Zhao^1^, MD; Yukui Ma^1^, MD; Bin Huang1*, MD; Zhoupeng Wu^1^, MD

^1^Affiliation: Department of Vascular Surgery, West China Hospital, Sichuan University, Chengdu, China

^2^Affiliation: Department of Cardiology, West China Hospital, Sichuan University, Chengdu, China

^#^Y.X. and C.C. contributed equally to this work.

**Corresponding author.** Bin Huang, MD. Department of Vascular Surgery, West China Hospital, Sichuan University, 37 Guo Xue Alley, Chengdu 610041, Sichuan Province, China. xgwkhb@126.com.

**Supplementary Materials - Index**

| **Supplementary Figures and Tables** |  |
| --- | --- |
| Table S1 | *pag. 2* |
| Table S2  Figure S1  Figure S2  Figure S3  Figure S4 | *pag. 3*  *pag. 4*  *pag. 5*  *pag. 6*  *pag. 7* |
|  |  |

**Supplementary Figures and Tables**

**Table S1. Search strategy**

| PubMed. Date: July 15 2022 | | Studies found |
| --- | --- | --- |
| #1 | ((Prostar) OR (Proglide)) OR (Perclose) | 365 |
| #2 | (((Suture based) OR (Vascular closure)) OR (Artery closure)) OR (Arteriotomy closure) | 28348 |
| #3 | (Aortic valve replacement) OR (Aortic valve implantation) | 44761 |
| #4 | ((#1) AND (#2)) AND (#3) | 73 |
| #5 | ((#4) AND (("2002"[Date-Publication]: "3000"[Date-Publication]))) AND (English[Language]) | 70 |
| Embase. Date: July 15 2022 | | Studies found |
| #1 | 'prostar'/exp OR prostar OR 'proglide'/exp OR proglide OR 'perclose'/exp OR perclose | 5851 |
| #2 | ('suture'/exp OR suture) AND based OR (vascular AND closure) OR (('artery'/exp OR artery) AND closure) OR (('arteriotomy'/exp OR arteriotomy) AND closure) | 43051 |
| #3 | aortic AND ('valve'/exp OR valve) AND ('replacement'/exp OR replacement) OR (aortic AND ('valve'/exp OR valve) AND ('implantation'/exp OR implantation)) | 74707 |
| #4 | #1 AND #3 AND #4 AND [2002-2022]/py AND [english]/lim | 651 |
| Cochrane library. Date: July 15 2022 | | Studies found |
| #1 | ((Prostar) OR (Proglide)) OR (Perclose) with Cochrane Library publication date from Jan 2002 to Apr 2022 | 64 |
| #2 | (((Suture based) OR (Vascular closure)) OR (Artery closure)) OR (Arteriotomy closure) | 2898 |
| #3 | (Aortic valve replacement) OR (Aortic valve implantation) | 2344 |
| #4 | ((#1) AND (#2)) AND (#3) | 13 |

**Table S2. Baseline characteristics of included patients comparing ProGlide and Prostar on vascular complications**

Data are described as n (%), mean ± stand deviation (SD), or median (interquartile range). Continuous variables were compared with the T-test. Categorical variables were analyzed using χ^2^ tests. ^*^ p<.05, ^**^ p<.01,^***^ p<.001. ^†^ The risk scores were calculated with EuroSCORE II (Dimitriadis, Berti, Heitzinger), Log EuroSCORE (Mehilli, Power), STS score mortality (Barbanti, Barbash, Marcusohn), and STS score mortality and morbidity (Seeger). NA: not available

|  |  | Age (years) | | Female gender | | Body mass index (kg/m2) | | Hypertension | | Diabetes mellitus | | Peripheral vascular disease | | Risk scores^†^ | |
| --- | --- | --- | --- | --- | --- | --- | --- | --- | --- | --- | --- | --- | --- | --- | --- |
| First author | Year | ProGlide | Prostar | ProGlide | Prostar | ProGlide | Prostar | ProGlide | Prostar | ProGlide | Prostar | ProGlide | Prostar | ProGlide | Prostar |
| Barbanti | 2015 | 80.6±8.8 | 80.6±5.2 | 78(62.4) | 83(54.2) | NA | NA | 102(81.6) | 125(81.7) | 37(29.6) | 54(35.3) | 13(10.4) | 13(8.6) | 6.1±4.6 | 5.4±3.6 |
| Barbash | 2015 | 81.5±8.7 | 81.6±6.0 | 231 (48.9) | 230 (48.7) | 26.6±5.5 | 26.3±4.6 | 375 (79) | 378 (80) | 144 (31) | 138 (29) | 83 (18) | 77 (16) | 8.8±6.3 | 8.3±6.1 |
| Mehilli | 2016 | 80.7±7.3 | 81.3±7.1 | 291(57.5) | 288(55.8) | 26.5±5.2 | 26.3±4.8 | NA | NA | 125(24.7) | 119(23.1) | 55(10.9) | 54(10.5) | 21.2±14.0 | 21.9±12.2 |
| Seeger | 2016 | 80.7±5.9 | 80.9±6.6 | 176(50.6) | 135(57.0) | 26.9±4.7 | 26.4±4.8 | NA | NA | 100(28.7) | 72(30.4) | NA | NA | 28.5±12.2 | 30.9±12.2 |
| Dimitriadis | 2017 | 82.6±5.0 | 82.8±5.8 | 103(56.3) | 130(60.5) | 26.0±4.6 | 26.1±4.2 | 119(65.0)^**^ | 162(75.3) | 43(23.5) | 62(28.8) | 16(8.7) | 20(9.3) | 6.0±5.6 | 7.1±6.7 |
| Power | 2019 | 82.2±6.7 | 82.7±6.3 | 197(50.0) | 169(48.0) | NA | NA | NA | NA | 116(29.4) | 104(29.5) | 64(16.2) | 49(13.9) | 17.3±10.4 | 16.6±10.0 |
| Berti | 2020 | 84.5±6.1 | 83.4±5.2 | 787(57.8) | 704(57.6) | 26.18±4.6 | 26.36±4.2 | NA | NA | 354(26.0) | 361(29.5) | NA | NA | 8.88±7.3 | 5.81±3.9 |
| Heitzinger | 2022 | 82(78,86) | 84(81,87) | 366(54.6) | 58(51.8) | 26.1(23.4,29.4) | 25.1(22.6,28.4) | 587(88) | 102(91) | 207(31) | 35(31) | 68(10) | 13(12) | 4.1(3.9,4.5) | 4.1(3.8,4.4) |
| Marcusohn | 2022 | 80(74,84) | 81(75,86) | 43(50.6) | 59(55.7) | 29.7(25.2,33.4) | 28.7(24.9,33.3) | 69(81.2) | 93(87.7) | 50(58.8)^***^ | 37(34.9) | 27(37.8)^***^ | 11(10.4) | 3.7(2.2,6.7) | 3.5(2.3,5.8) |

**
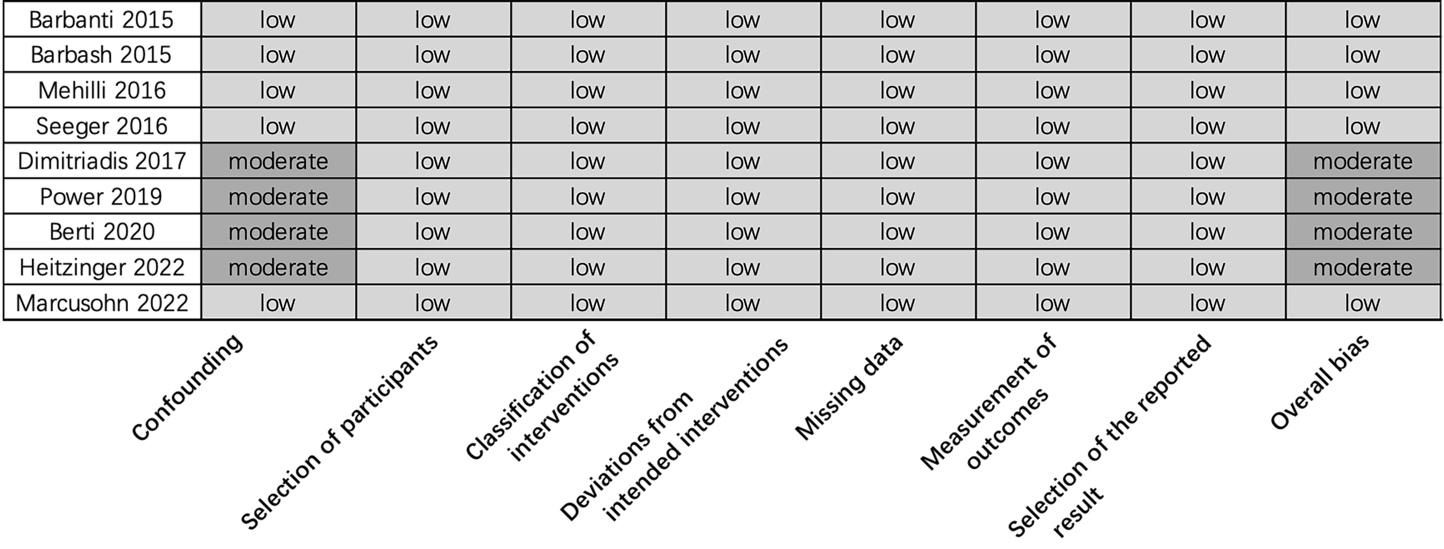
**

Figure S1. The risk of bias in each study assessing by the Risk of Bias In Non-Randomized Studies of Interventions (ROBINS-I) tool.


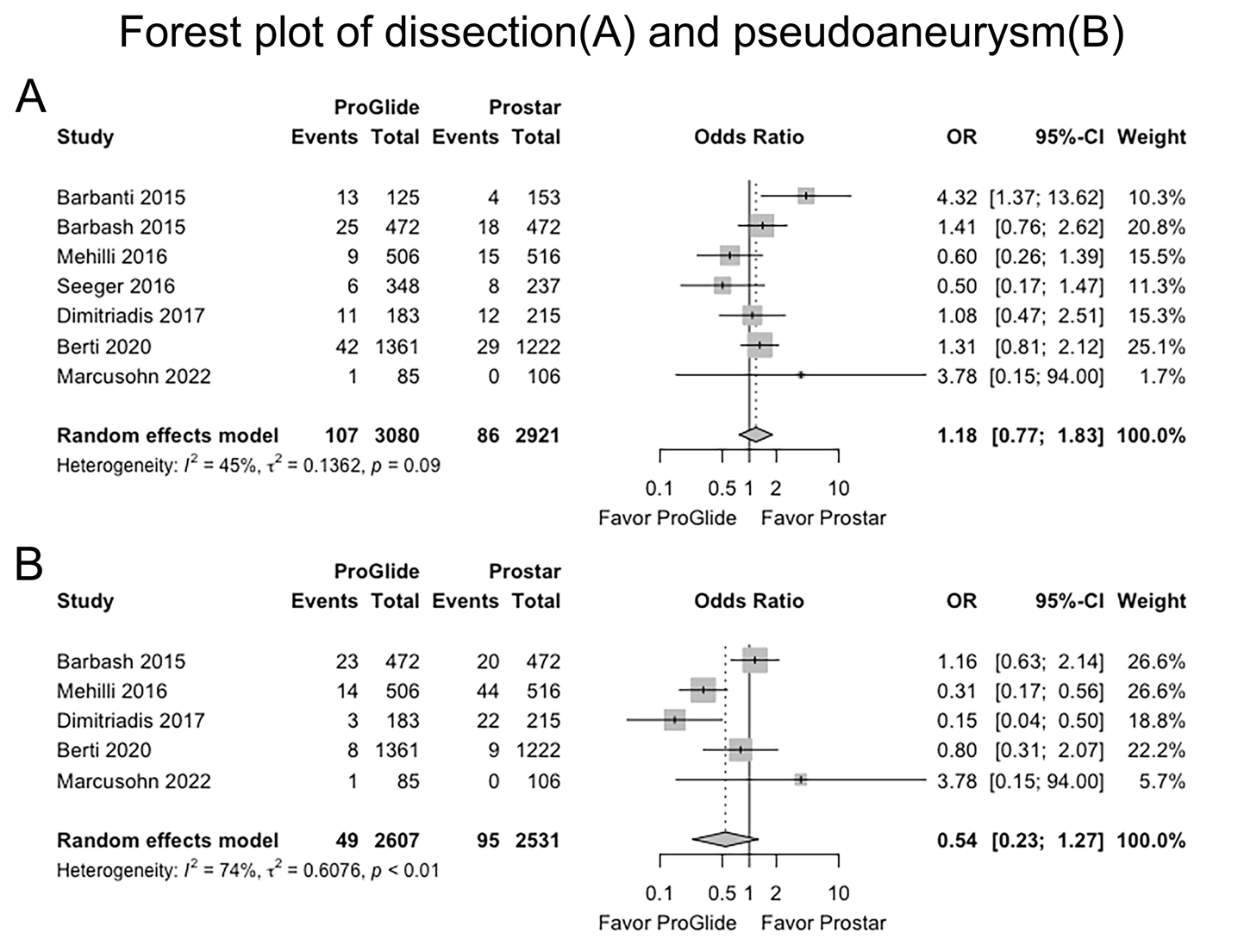


Figure S2. Forest plot of (A) 7 studies reporting the risk of dissection and (B) 5 studies reporting the risk of pseudoaneurysm. OR = odds ratio; CI = conﬁdence interval.


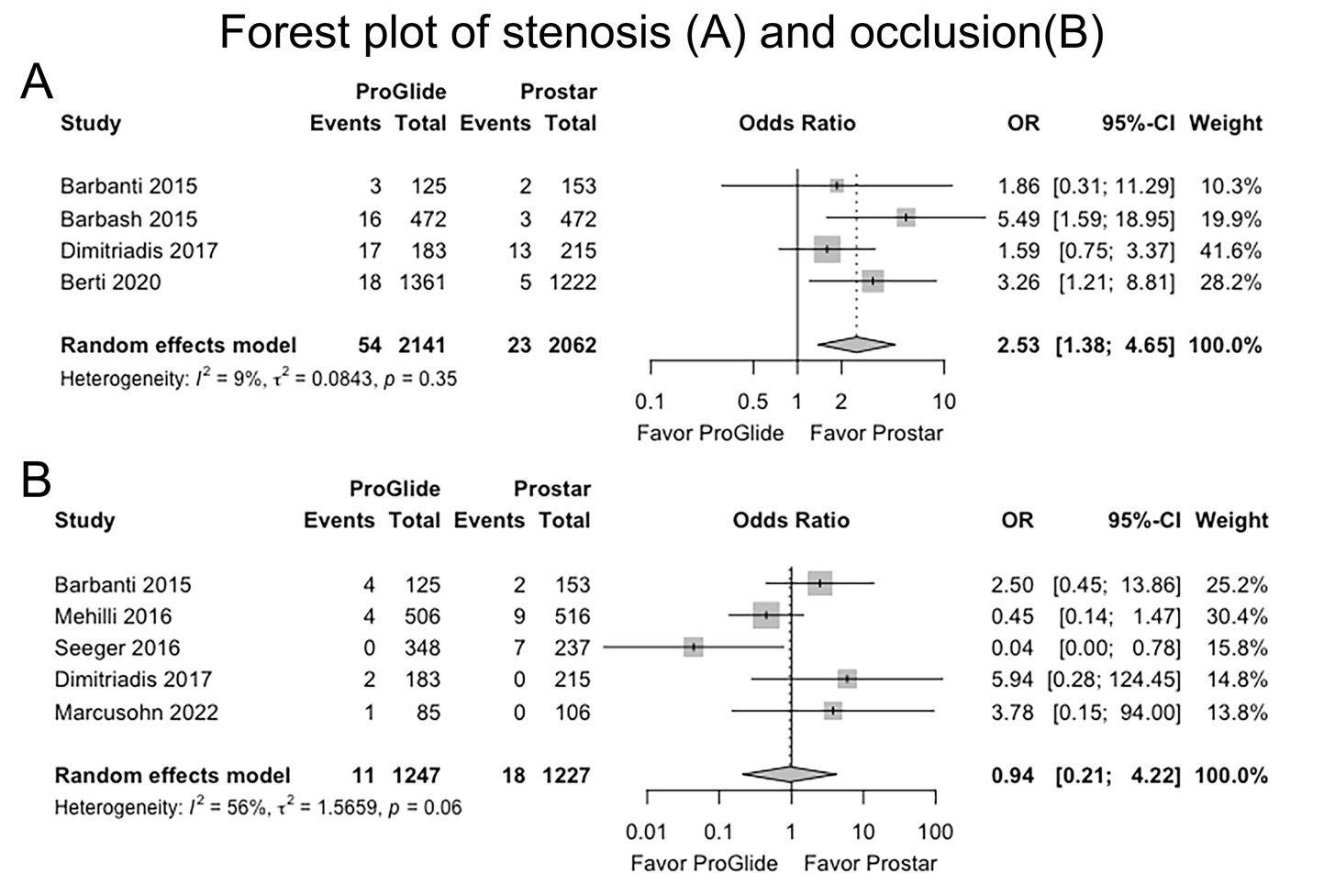


Figure S3. Forest plot of (A) 4 studies reporting the risk of stenosis and (B) 5 studies reporting the risk of occlusion. OR = odds ratio; CI = conﬁdence interval.


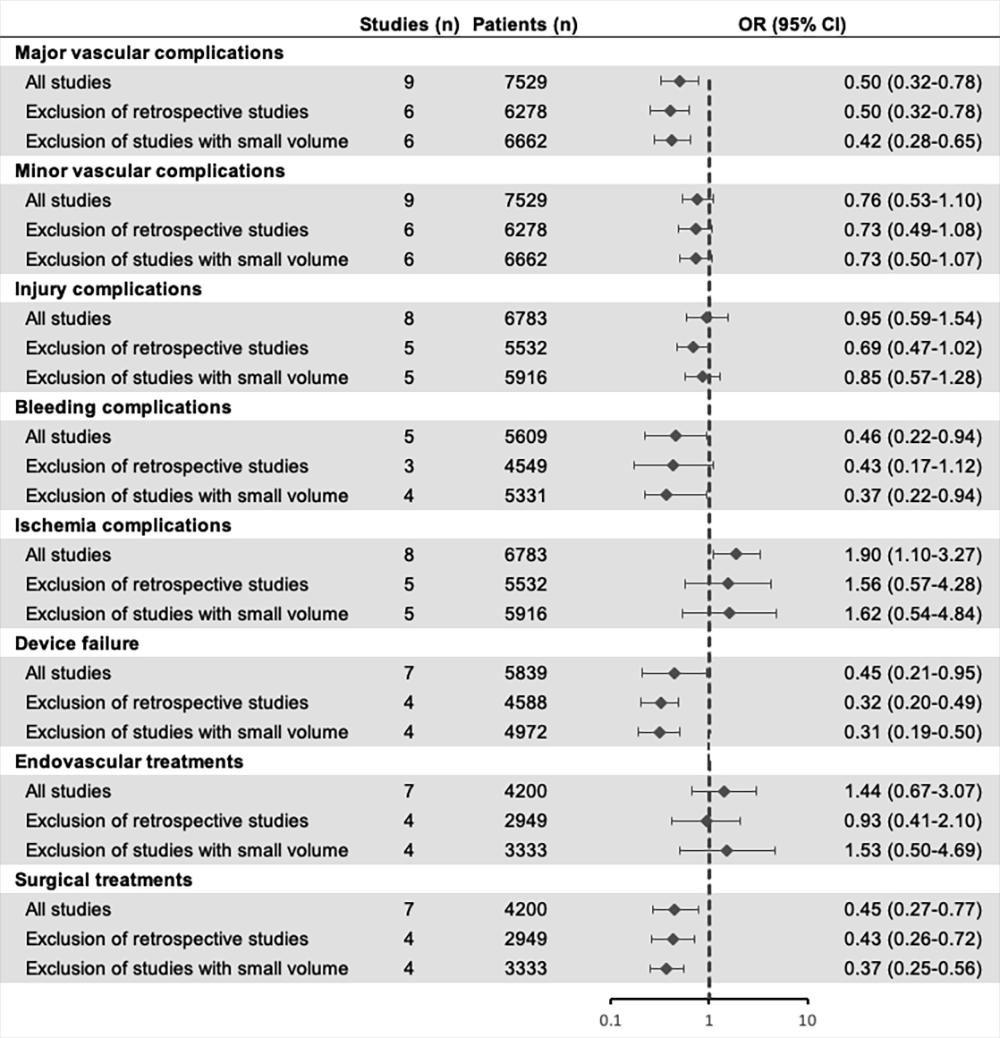


Figure S4. Sensitivity analyses of the 9 studies on outcomes with the exclusion of retrospective or small-volume studies (patients less than 500). OR = odds ratio; CI = conﬁdence interval.
